# Multimorbidity as assessed by reporting of multiple causes of death: variations by period, sociodemographic characteristics and place of death among older decedents in England and Wales, 2001–2017

**DOI:** 10.1136/jech-2021-217846

**Published:** 2022-06-02

**Authors:** Emily Marjatta Grundy, Rachel Stuchbury

**Affiliations:** 1 Institute for Social & Economic Research, University of Essex, Colchester, UK; 2 CeLSIUS, EPH, University College London, London, UK

**Keywords:** AGING, DEATH CERTIFICATES, Health inequalities, LONGITUDINAL STUDIES, RECORD LINKAGE

## Abstract

**Background:**

Multimorbidity is common at older ages and is associated with disability, frailty and poor quality of life. Research using clinical databases and surveys has shown associations between multimorbidity and indicators of social disadvantage. Use of multiple coded death registration data has been proposed as an additional source which may also provide insights into quality of death certification.

**Methods:**

We investigate trends in reporting multiple causes of death during 2001–2017 among decedents aged 65 years and over included in a census-based sample of 1% of the England and Wales population (Office for National Statistics Longitudinal Study). Using Poisson regression analysis, we analyse variations in number of mentions of causes of death recorded by time period, place of death, age, sex and marital status at death and indicators of health status and individual and area socioeconomic disadvantage reported at the census prior to death.

**Results:**

Number of mentions of causes recorded at death registration increased 2001–2017, increased with age, peaking among decedents aged 85–9 years, and was positively associated with indicators of prior disadvantage and poor health, although effects were small. Number of mentions was highest for hospital decedents and similar for those dying in care homes or their own homes.

**Conclusion:**

Socioeconomic disadvantage, prior poor health, dying in hospital and older age—although not extreme old age—are associated with dying with more recorded conditions. Results may reflect both differences in multimorbidity at death and variations in quality of medical certification of death. Quality of death certification for decedents in care homes needs further investigation.

**WHAT IS ALREADY KNOWN ON THIS TOPIC**

- Use of multiple cause of death information has been proposed as a means of assessing multimorbidity at time of death. Recording of multiple causes of death reported in studies from France, Italy and the USA show similar increases in number of mentions with older age to other types of study; the highest number of mentions are for hospital decedents and the lowest number are for those dying in their own homes.

**WHAT THIS STUDY ADDS**

- We use nationally representative data for a 17-year period from a record linkage study which includes information both from death registration data and from study members’ prior census returns, includes the care home population and is large enough to allow disaggregation of the oldest age groups.

**HOW THIS STUDY MIGHT AFFECT RESEARCH, PRACTICE AND/OR POLICY**

- Number of mentions was highest for hospital decedents but, unlike results from US and Italian studies, was similar for decedents in care homes and private residences, despite high levels of multimorbidity in the care home population. This suggests that the quality of medical certification of deaths among care home decedents in England and Wales needs further investigation, especially as the proportion of deaths in this setting is increasing.

## Introduction

The greater availability of life-prolonging treatments and associated older ages at death mean that to an increasing extent death results from a combination of diseases, rather than a single pathological process.^1^ Multimorbidity, defined as the coexistence of two or more long-term conditions,^2^ is associated with increased disability, poor quality of life and high healthcare use and was recognised as an inadequately understood challenge even before the COVID-19 pandemic further emphasised associated elevated risks of mortality.^3^ Research on multimorbidity has predominantly been based on analyses of clinical databases^4–17^ or surveys.^18–21^ Use of multiple coded cause of death (MCoD) data has been proposed as an additional source which may also provide insights into quality of cause of death coding, with a suggestion that a higher number of reported mentions indicates better reporting.^22 23^ We use data from a nationally representative census-based record linkage study of England and Wales to investigate associations between recording of multiple causes of death and sociodemographic characteristics recorded at death and reported by study members at the population census prior to death. We also compare trends in number of causes of death recorded over the period 2001–2017.

### Previous research

Studies of multimorbidity have used diverse measures and definitions precluding direct comparisons of results.^2^ A common finding is of strong associations between multimorbidity and older age, although some plateauing or decline in prevalence after age 80 or 85 years has been reported in the few studies which present results for the oldest groups.^10 11^ Some studies report a higher prevalence of multimorbidity among women^4 8 10 13 15 17 19^ but others find no sex differences^5 6 11 12^ or a higher prevalence among men.^7^ Several studies have reported associations between multimorbidity and indicators of disadvantage,^24^ measured at the area^4–6 9^ or individual^7 8 15 17–19^ level. Differentials by household status have rarely been considered and some studies exclude residents of institutions^7 8 10 18–20^ or do not state whether they are included.^6 9–14 16 17^ One study based on Netherlands primary care records for the early 1990s reported higher levels of multimorbidity for those living alone or in care homes rather than those living with a spouse or other family members.^15^ A more recent prospective study of Finnish nonagenarian found that multimorbidity was associated with long-term care admission.^21^ Increases in age-specific prevalence rates of multimorbidity have been reported in some studies, hypothesised to reflect adverse changes in lifestyles and improvements in ascertainment and treatment of some conditions.^8 25 26^ Studies of number of recorded causes of death among decedents report similar variations by age to assessments from clinical database and survey data.^22 27–31^ Grippo *et al* 31 found that among decedents aged 50 years and over in Italy recording of multiple causes of death peaked at ages 85–9 years. However, unlike some results from other studies, analyses based on death certificate data indicate a higher number of causes reported for men than women.^27–29 31^ Differentials by marital status and place of death have also been reported. Wall *et al* 23 found that recording multiple causes of death in Minnesota was higher for the non-married than the married; highest for decedents in hospitals; and higher for nursing home decedents than for those dying at home. A more recent study based on French and Italian data found fewer causes reported for the never married and more causes recorded for those dying in hospital, and in Italy also for those dying in homes for older people, than for those dying in their own homes.^27^

### Current study

These previous studies using MCoD approaches to investigate multimorbidity have generally been limited to considering information recorded at death. We also consider individual characteristics reported by study members at the population census prior to death. We expected that number of causes recorded would increase over the time period considered due to diagnostic advances and longer survival of those with multiple conditions as well as increases in multimorbidity reported in some studies. Based on the previous literature, we expected that number of mentions would be positively associated with older age, although possibly with some drop back in the very oldest groups, and with indicators of socioeconomic disadvantage and prior poor health. We also expected numbers of causes recorded to be highest for hospital decedents, reflecting their higher morbidity and greater use of diagnostic tests. Residents in care homes also have high and increasing levels of multimorbidity,^21 32^ so we also expected them to have a higher number of conditions recorded compared with those dying at home.

## Methods

We use data from the Office for National Statistics Longitudinal Study (ONS LS),^33^ a census-based multicohort record linkage study of a 1% representative sample of the population of England and Wales. The initial sample was drawn from the 1971 Census but has been continuously updated with the addition of immigrants with an LS birthday and individual level data from subsequent censuses linked to vital registration records. This analysis is based on deaths at ages 65 years and over in 2001–2017 among LS sample members aged 55 years and over at the 2001 Census and/or aged 65 years and over at the 2011 Census. 2011 Census data were missing for 9.8% of the study population not recorded as having died or emigrated by this date. These study members were necessarily excluded from analyses including 2011 Census data but are included in analyses based solely on death registration data. Reasons for missing census data include non-completion of a census form, unrecorded emigration or record linkage failure. In a few cases (<1%), study members had missing data for specific variables of interest and were excluded from analyses using those variables. Data were accessed in the ONS safe setting and were fully anonymised and outputs were subject to data clearance protocols.

### Measures

The outcome measure, number of causes of death recorded, was drawn from the Medical Certificate of Cause of Death which includes underlying cause of death (UCD) and, in the ONS LS, up to eight additional mentions of causes recorded as part of the causal sequence leading (Part 1 of death certificate) or contributing to death (Part 2). Deaths were coded using the International Statistical Classification of Diseases and Health Related Problems, 10th Revision (ICD-10) using three-digit or, in the case of more diverse groupings, four-digit codes. We counted as additional causes of death all mentions which had a different three-digit or, where applicable, four-digit code from the UCD. ONS introduced ICD-10 V201 in January 2011 and in January 2014 changed the automatic coding software death to IRIS, which incorporates official updates to ICD-10 approved by the WHO. These changes involved minor amendment of modification and selection rules for ascertaining a causal sequence which influenced assignment in some cause groups (including dementia) but would not have affected number of conditions reported.^34^

Information on place of death and age, sex and marital status at death was drawn from death registration data. We grouped place of death into three categories: hospital, including the small proportion dying in hospices; nursing, residential or other type of care home or communal establishment (henceforth referred to as care homes); and private residences (the very small number of deaths occurring elsewhere, eg, on roads, was included in this category). We used linked data from study members’ last census record prior to death (2001 or 2011) to capture information on prior sociodemographic and health characteristics. These included self-rated health; presence of a long-term illness that limited activities; a derived combined indicator of housing tenure and household type (owner occupier; renter; resident in a care home); and an indicator of whether participants had a postsecondary educational qualification. In the 2001 Census, questions on educational qualifications were not asked of adults aged 75 years and over; so for those older than that who died before the 2011 Census, we drew information from their earlier census records, where available. We additionally included an indicator of area deprivation based on ward level Carstairs quintile.^35^

### Analysis strategy

In analyses including only information collected at death, we consider three time periods: from the 2001 Census (20 April 2001) to the end of 2005; from 2006 to the 2011 Census (27 March 2011); and from the 2011 Census to the end of 2017, to investigate changes in reporting of additional causes of death over time. Descriptive information on variation in number of causes of death recorded by place of death is presented for the most recent period (2011–2017). In the main analysis including census characteristics, we focus on two periods of near equivalent length, from the 2001 Census to the end of 2007 and from the 2011 Census to the end of 2017. Many characteristics of interest are interrelated, for example, admission to and death in care homes are associated with being unmarried^36 37^ necessitating a multivariate approach. As the outcome is a count (number of mentions), we fitted multivariate Poisson models using robust standard errors. In sensitivity analyses, we also fitted negative binomial models to number of mentions in addition to the underlying cause which showed essentially the same results. Models based solely on death registration data included year of death and those including census variables an indicator of years since the relevant census to adjust for the trend towards increased number of mentions and the timeliness of the census information. Education was not included in the multivariate models as it was not significant in univariate analysis and preliminary analyses showed inclusion did not improve model fit.

## Results

### Trends 2001–2017 from death certification data only

**Figure 1:**
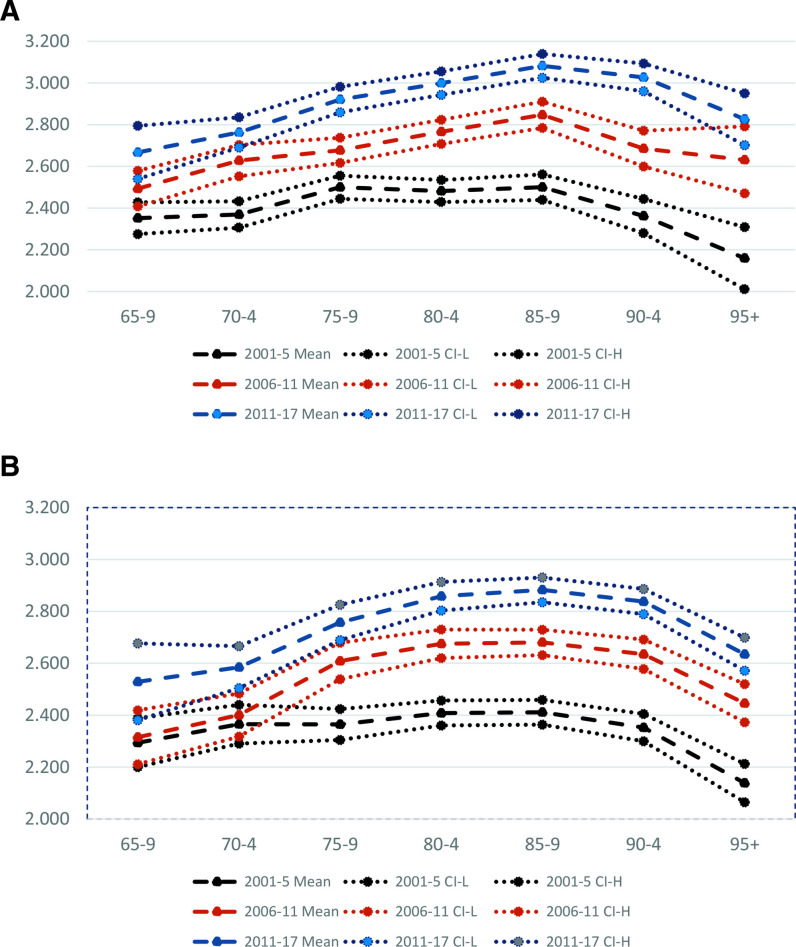
Mean (95% CI) number of causes of death recorded by period and age group at death England & Wales, (A) Men (B) Women. Source: analysis of Office for National Statistics Longitudinal Study.

**Figure 2:**
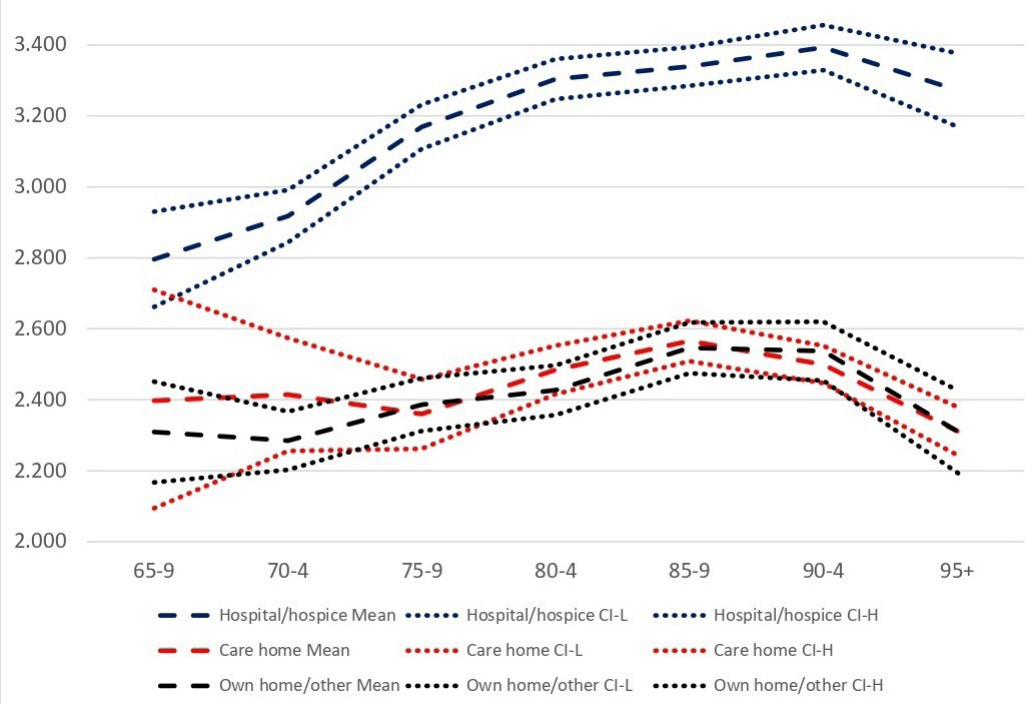
Mean (95% CI) number of causes of death by place of death and age group at death, England & Wales, 2011–17. Source: analysis of Office for National Statistics Longitudinal Study.

Over the period 2001–2017, 23.2% of decedents had no causes additional to the UCD recorded, 30.6% had two causes recorded, 22.8% had three and 23.6% had four or more. As shown in figure 1, the mean number of causes mentioned increased over the period considered. For male decedents aged 85–9 years in 2011–2017, for example, mean number of causes recorded was 3.1 (3.0–3.1) compared with 2.5 (2.4–2.6) in 2001–2005. In 2001–2005, mean number of causes recorded increased from age 65–9 to 70–4 years, plateaued between ages 75–9 and 85–9 years and then dropped; in 2006–2011 and 2011–2017, increases in mean numbers of causes were evident until age 85–9 years before falling back. As illustrated for the 2011–2017 period in figure 2, number of causes of death recorded was higher for those dying in hospital compared with those dying at home or in a care home, for whom number of reported causes was similar.

Results from multivariate Poisson analyses of number of causes (online supplemental appendix 1), including only variables recorded at death (5-year age group, place of death, sex, marital status at death, year of death), showed a positive but non-linear association between age at death and number of mentions, with the highest number recorded for decedents aged 85–9 years.

10.1136/jech-2021-217846.supp1 Supplementary data

Results also showed a lower incidence rate ratio for never-married and currently married women relative to widows. Mean number of causes of death recorded was higher for decedents in hospital than for those dying at home and slightly raised for male decedents in care homes. There was a positive association between later year of death and number of mentions.

### Variations in number of causes reported; census and linked death registration data 2001–2007 and 2011–2017

Table 1 shows the distribution of the sample by characteristics recorded at death and at the census preceding death. Some variations by period reflect cohort differences in educational attainment, housing tenure and marital history and improvements in mortality leading to a shift to older ages at death. For example, 27% of decedents in the later period were aged 90 years and over compared with 19% in 2001–2007.

**Table 1:** Distribution of the sample by characteristics recorded at death registration and at census prior to death; decedents aged 65 years and over 2001–2007 and 2011–2017, England and Wales

	2001–2007, n=30 259		2011–2017, n=31 417 (28 358 with 2011 Census data)	
	%	n	%	n
Sex				
M	44.92	13 593	45.54	14 308
F	55.08	16 666	54.46	17 109
Educational qualifications*				
Higher secondary+	10.51	3179	25.88	8132
Lower or none	89.49	27 080	74.12	23 285
Carstairs deprivation quintile				
1–2, least deprived	27.12	8161	29.50	8362
2	15.58	4714	17.18	4869
3	20.70	6265	21.53	6102
4	23.99	7258	23.06	6537
5, most deprived	27.79	8409	25.92	7347
Household type/tenure				
Owner	57.00	17 245	63.97	18 136
Renter	31.18	9434	25.69	7283
Care home†	11.81	3574	10.34	2931
Limiting long-term illness				
No	29.44	8909	21.30	6038
Yes	70.56	21 350	78.70	22 316
Self-rated heath				
Good	21.85	6612	26.54	7526
Fair	39.18	11 855	43.25	12 264
Poor	38.97	11 792	30.21	8568
Age at death (years)				
65–9	8.43	2549	3.20	1006
70–4	12.09	3658	10.50	3298
75–9	17.78	5379	15.63	4909
80–4	22.20	6715	21.00	6597
85–9	20.06	6067	23.07	7249
90–4	13.87	4195	18.37	5771
95+	5.57	1684	8.22	2583
Marital status at death				
Widowed	51.11	15 464	49.87	15 669
Married	35.94	10 875	35.63	11 193
Divorced/separated	4.96	1500	7.68	2414
Never married	8.00	2420	6.81	2141
Place of death				
Private home/other	17.73	5364	21.17	6650
Care home†	20.44	6186	25.89	8135
Hospital/hospice	61.83	18 709	52.94	16 632

Source: analysis of Office for National Statistics Longitudinal Study.

*Those with no information (5.6% 2001–2007; 1% 2011–2017) were treated as having no qualification.

†Or other type of communal establishment.

Table 2 presents mean (95% CI) number of causes of death recorded by these characteristics. Means are weighted by 5-year age group at death as some characteristics, for example, death in a care home, are strongly associated with age at death. Mean number of mentions was positively associated with living in a more deprived area, reporting long-term illness, reporting fair or poor self-rated health and, in 2011–2017, with being a renter rather than an owner occupier at the preceding census; however, those who had then lived in a care home had a lower mean number of mentions compared with those then living in private households. Fewer average mentions were reported for women who were never married at death compared with those of other marital statuses and number of mentions was highest for those dying in hospital.

**Table 2:** Mean (95% CI) number of causes of death recorded by period and characteristics at death registration and at census prior to death, weighted by 5-year age group at death; decedents aged 65 years and over 2001–2007 and 2011–2017, England and Wales

	2001–2007, n=30 247		P value	2011–2017, n=31 417		P value
	Mean	95% CI		Mean	95% CI	
Sex						
M	2.502	2.480 to 2.523		2.973	2.947 to 3.000	
F	2.403	2.383 to 2.422	^*^	2.796	2.773 to 2.819	^*^
Educational qualifications†						
Higher secondary+	2.450	2.403 to 2.498		2.838	2.804 to 2.872	
Lower or none	2.439	2.424 to 2.455		2.884	2.864 to 2.905	
Carstairs deprivation quintile						
1–2, least deprived	2.385	2.358 to 2.413		2.791	2.758 to 2.824	
3	2.414	2.383 to 2.446		2.822	2.783 to 2.861	
4	2.466	2.436 to 2.496	^*^	2.898	2.859 to 2.936	^*^
5, most deprived	2.508	2.480 to 2.537		3.013	2.976 to 3.050	
Household type/tenure						
Owner	2.450	2.431 to 2.469		2.878	2.854 to 2.901	
Renter	2.481	2.455 to 2.508		3.007	2.969 to 3.045	^*^
Care home‡	2.338	2.299 to 2.377	^*^	2.597	2.547 to 2.646	^*^
Limiting long-term illness						
No	2.354	2.327 to 2.381		2.661	2.621 to 2.700	
Yes	2.479	2.462 to 2.497	^*^	2.931	2.910 to 2.952	^*^
Self-rated heath						
Good	2.347	2.317 to 2.378		2.690	2.654 to 2.725	
Fair	2.433	2.410 to 2.456	^*^	2.916	2.888 to 2.944	^*^
Poor	2.510	2.487 to 2.534 ^*^	^*^	2.987	2.953 to 3.021	^*^
Marital status at death						
Widowed	2.437	2.417 to 2.457		2.867	2.843 to 2.891	
Married	2.476	2.451 to 2.501		2.907	2.877 to 2.937	
Divorced/separated	2.426	2.362 to 2.490		2.900	2.835 to 2.964	
Never married	2.383	2.333 to 2.433		2.730	2.668 to 2.793	^*^
Place of death						
Private home/other	2.209	2.181 to 2.380		2.440	2.408 to 2.471	
Hospital/hospice	2.585	2.565 to 2.605	^*^	3.265	3.239 to 3.291	^*^
Care home	2.243	2.216 to 2.270		2.467	2.439 to 2.495	
All	2.444	2.430 to 2.459		2.873	2.856 to 2.891	^*^

Source: analysis of Office for National Statistics Longitudinal Study.

*p<0.05 for difference from reference category (first listed).

†Those with missing data (5.6% 2001–2007; 1% 2011–2017) were treated as having no qualification.

‡Or other type of communal establishment.

### Multivariate analyses

Results from Poisson regression analyses (table 3) showed that among male decedents having reported long-term illness at the last census and fair or poor, rather than good, health were positively associated with number of mentions. In 2011–2017, living in an area in one of the two most deprived quintiles, rather than one of the two least deprived, and having been a renter rather than an owner-occupier in 2011 were both positively associated with number of mentions. In 2001–2007, dying at ages 75–89 years was associated with a higher and dying at ages 95 years and over was associated with a lower number of reported causes compared with dying at age 65–9 years. In 2011–2017, decedents aged 75–94 years had a higher number of mentions compared with those dying at ages 65–9 years. Death in hospital was positively associated with number of causes recorded. Results for women were similar although the effect of having been a renter rather than an owner-occupier at the census prior to death was only evident in analyses for both periods combined.

**Table 3:** Results from Poisson regression models (incidence rate ratios (IRRs) and 95% CIs) of number of causes of death by characteristics at census prior to death and at death; decedents aged 65 years and over 2001–2007 and 2011–2017, England and Wales

Men	2001–2007, n=13 520			2011–2017, n=13 026		
	IRR	95% CI	P value	IRR	95% CI	P value
Carstairs deprivation quintile; Ref. 1–2 (least deprived)						
3	1.008	0.983 to 1.034		1.015	0.989	
4	1.015	0.990 to 1.040		1.029	1.003 to 1.055	^*^
5	1.014	0.989 to 1.039		1.055	1.029 to 1.081	***
Household type/tenure; Ref. Owner						
Renter	1.001	0.981 to 1.022		1.026	1.003 to 1.049	^*^
Care home†	1.041	1.002 to 1.081	^*^	1.019	0.980 to 1.061	
Limiting long-term illness; Ref. No						
Yes	1.060	1.036 to 1.085	***	1.072	1.043 to 1.101	***
Self-rated heath; Ref. Good						
Fair	1.047	1.021 to 1.073	***	1.051	1.024 to 1.078	***
Poor	1.076	1.046 to 1.107	***	1.081	1.049 to 1.113	***
Age group at death (years); Ref. 65–9						
70–4	1.023	0.987 to 1.061		1.014	0.958 to 1.074	
75–9	1.068	1.033 to 1.104	***	1.063	1.006 to 1.123	^*^
80–4	1.064	1.028 to 1.100	***	1.101	1.042 to 1.162	**
85–9	1.074	1.037 to 1.113	***	1.128	1.068 to 1.192	***
90–5	0.996	0.955 to 1.038		1.115	1.053 to 1.181	***
95+	0.929	0.868 to 0.994	^*^	1.061	0.989 to 1.137	
Marital status at death; Ref. Widowed						
Married	0.984	0.964 to 1.005		0.998	0.976 to 1.020	
Divorced/separated	0.999	0.958 to 1.042		1.005	0.967 to 1.045	
Never married	0.972	0.938 to 1.006		0.982	0.943 to 1.014	
Place of death; Ref. Private home/other						
Hospital/hospice	1.152	1.128 to 1.176	***	1.312	1.283 to 1.341	***
Care home	1.041	1.010 to 1.073		0.991	0.961 to 1.021	
Period Ref. 2001–2007	1.00			1.201	1.185 to 1.216	***
**Women**	**2001–2007, n=16 561**			**2011–2017, n=15 305**		
Carstairs deprivation quintile; Ref. 1–2 (least deprived)						
3	1.006	0.982 to 1.029		0.994	0.970 to 1.006	
4	1.033	1.010 to 1.057	**	1.008	0.984 to 1.032	
5	1.043	1.020 to 1.066	***	1.032	1.008 to 1.056	^*^
Household type/tenure; Ref. Owner						
Renter	1.009	0.990 to 1.028		1.019	0.998 to 1.040	
Care home†	1.017	0.991 to 1.044		0.988	0.961 to 1.017	
Limiting long-term illness; Ref. No						
Yes	1.057	1.033 to 1.081	***	1.092	1.061 to 1.125	***
Self-rated heath; Ref. Good						
Fair	1.012	0.989 to 1.036		1.075	1.048 to 1.103	***
Poor	1.049	1.024 to 1.075	***	1.122	1.091 to 1.154	***
Age group at death (years); Ref. 65–9						
70–4	1.022	0.977 to 1.068		0.999	0.934 to 1.069	
75–9	1.038	0.995 to 1.083		1.063	0.997 to 1.134	
80–4	1.055	1.013 to 1.098	**	1.114	1.046 to 1.186	**
85–9	1.057	1.015 to 1.101	**	1.127	1.058 to 1.200	***
90–5	1.049	1.005 to 1.094	^*^	1.126	1.056 to 1.200	***
95+	0.967	0.921 to 1.015		1.075	1.005 to 1.148	^*^
Marital status at death; Ref. Widowed						
Married	1.007	0.984 to 1.030		0.985	0.962 to 1.008	
Divorced/separated	0.952	0.916 to 0.990		0.995	0.961 to 1.030	
Never married	0.989	0.960 to 1.020		0.963	0.928 to 1.000	
Place of death; Ref. Private home/other						
Hospital/hospice	1.167	1.141 to 1.193		1.348	1.317 to 1.380	***
Care home	1.004	0.977 to 1.031		1.018	0.990 to 1.046	
Period; Ref. 2001–2007	1.00			1.182	1.168 to 1.197	***

Source: analysis of Office for National Statistics Longitudinal Study. Models also include years since census.

*p<0.05; **p<0.01, ***p<0.001.

†Or other type of communal establishment.

## Discussion

Strengths of this study include use of nationally representative data for a large sample for a 17-year period including information recorded at death and decedents’ own reports of health and circumstances at the population census prior to death. Residents of care homes were included and explicitly examined, whereas many studies have excluded this group or not reported variations in multimorbidity by household type. The study has, however, several limitations. Census data were missing for some 10% of the 2011 Census sample and ONS has estimated an undercount of 6% in the 2001 Census.^38^ This may be a source of slight bias but these inclusion rates are much higher than in surveys which have been used to examine multimorbidity^18–20^ and probably equivalent to or higher than linkage rates in clinical databases which are rarely reported. A more important limitation is that sociodemographic characteristics may be associated both with differentials in multimorbidity and with variations in quality of recording cause of death.^39^ Zellweger *et al*,^30^ for example, used Swiss National Cohort data for 2010–2012 to compare reported causes of death with hospital discharge diagnoses at death and found that concordance was lower for older age groups, the socially disadvantaged and the never married. Similar limitations may apply to ascertainment of multiple morbidity using other sources due to variations in seeking healthcare and the quality of recording of conditions. A study of multimorbid patients in Germany, for example, found that concordance between self-reported and general practitioner-reported chronic conditions was poorer for patients with lower levels of education.^40^ Additionally, we only considered number of mentions of causes of death, rather than constellations of diseases, and make an implicit assumption, as have previous investigators,^22 23^ that recording more causes of death is associated with better death certification quality. This assumption needs further investigation

Results showed an increase in number of causes recorded over time. This is consistent with findings from the few studies which have examined trends in multimorbidity and reported increases over and above those due to population ageing.^8 25 26^ This is clearly an important public health concern, although how much of this increase is due changes in morbidity profiles and how much to changes in investigations and diagnoses is as yet unclear. It is also possible that the increased focus on medical certification of death in the inquiries following the Shipman and other scandals and consultations on establishment of a medical examiner system^41^ may also have influenced certification practices. Mean number of causes and variations by age and sex were similar to those reported in recent studies based on death certificate data.^27–31^ The peak in number of causes recorded at age 85–9 years in the more recent period considered is also consistent with results from those studies based on clinical databases which present results for the oldest age groups.^10 11^ It has not been established whether the slight downturn in recorded multimorbidity in those studies and in number of causes of death in this study reflects less multimorbidity, due to a selective survival effect, or less rigorous investigation and ascertainment of conditions. This merits further investigation. We also found associations between census-based indicators of disadvantage and poorer health and a higher number of recorded causes of death, consistent with the higher burden of multimorbidity in less advantaged groups reported in other types of study,^4–7^ however effects were small.

Studies from other countries based on MCoD data have reported a higher number of mentions for decedents in hospital and, in some cases, also for people dying in nursing and care homes, compared with those dying at home.^23 28^ Our results similarly show the highest number of mentions for hospital decedents. However, we found little difference in mentions between those dying in their own homes and those dying in care homes despite high and increasing levels of multimorbidity in the care home population^32^ and the large proportion of care home residents with dementia among whom levels of multimorbidity are higher than for those with other conditions.^42–44^ Investigating the specific role of deaths attributed to dementia and number of causes reported was beyond the scope of this paper and would be complicated by needing to allow both for a trend towards greater reporting of dementia^37^ and changes in coding protocols.^34^ However, over the whole period considered, the data we used showed that among decedents for whom dementia or Alzheimer’s disease was recorded as an underlying or contributing cause of death, 67% of those who died in a care home had only one or two causes mentioned compared with 55% of those dying at home and 51% of those dying in hospital. This suggests a need to focus more attention on cause of death recording for decedents in care homes, especially as the proportion of deaths in this setting is increasing,^37^ particularly for those with dementia who comprise a large component of the care home population.

Inadequacies in death certification practice are well recognised^1^ but medical certification of death provides essential information on the epidemiological profile of the population and the COVID-19 pandemic—as well as in the UK, the Shipman and other scandals—has emphasised the need for accurate and scrutinised recording. This study demonstrates the potential of linked death certification and census data to inform investigation of trends and differentials in multimorbidity which is recognised as a poorly understood and growing challenge. The new medical examiner system in England and Wales is currently being rolled out in a geographically phased way.^45^ Future analyses of the data we use here, which will soon be augmented by inclusion of 2021 Census data, including analyses by region and for other subgroups, may be useful in assessing any impact on multiple cause of death recording.

## Data Availability

Data may be obtained from a third party and are not publicly available. Office for National Statistics (ONS) allows research access to the ONS Longitudinal Study in controlled conditions.
