# Supplementary material for: Multimorbidity as assessed by reporting of multiple causes of death: variations by period, sociodemographic characteristics and place of death among older decedents in England and Wales, 2001–2017
Source: J Epidemiol Community Health. 2022 Jun 2;76(8):699–706. doi: 10.1136/jech-2021-217846 (PMC9279827; doi:10.1136/jech-2021-217846)
Supplement: Supplementary data [file jech-2021-217846supp001.pdf]

Appendix Table 1: Results from Poisson regression models (Incidence Rate Ratios and 95% confidence intervals) of number of causes of death recorded by sex, age group and marital status at death and place of death, decedents aged 65 and over 2001-17, England & Wales.

At death:	**Men n= 34,316**				**Women n=41,577**				**Persons n=75,893**			
**Sex**	IRR	95% CI			IRR	95% CI			IRR	95% CI		
**Female**									0.954	0.947	0.962	***
**Age group**												
65-9	1.00				1.00				1.00			
70-4	1.017	0.991	1.045		1.013	0.980	1.047		1.016	0.995	1.037	
75-9	1.061	1.035	1.087	***	1.069	1.036	1.103	***	1.064	1.043	1.084	***
80-4	1.082	1.056	1.109	***	1.107	1.075	1.141	***	1.093	1.072	1.113	***
85-9	1.109	1.082	1.137	***	1.118	1.085	1.152	***	1.111	1.090	1.132	***
90-5	1.076	1.047	1.107	***	1.116	1.082	1.151	***	1.097	1.075	1.119	***
95+	1.019	0.980	1.059		1.049	1.015	1.085	**	1.034	1.010	1.058	**
**Marital status**												
Widowed	1.00				1.00				1.00			
Married	0.989	0.976	1.002		0.985	0.971	0.999	*	0.984	0.975	0.994	**
Div/sep.	1.012	0.987	1.037		0.978	0.956	1.001		0.993	0.977	1.010	
Never-married	0.992	0.970	1.014		0.963	0.944	0.983	***	0.977	0.963	0.992	**
**Place of death**												
Home/other	1.00				1.00				1.00			
Hospital/hospice	1.247	1.230	1.265	***	1.264	1.246	1.282	***	1.255	1.243	1.267	***
Care home	1.031	1.012	1.050	**	1.015	0.999	1.031		1.019	1.007	1.032	**
**Year of death (ref.2001)**	1.018	1.017	1.019	***	1.017	1.016	1.018	***	1.017	1.016	1.018	***

***p<0.001.**p<0.01; *p<0.05

Source: Analysis of ONS Longitudinal Study

Grundy EM, Stuchbury R. *J Epidemiol Community Health* 2022; 76:699–706. doi: 10.1136/jech-2021-217846
